# Editorial—Focus on tibia shaft fractures

**DOI:** 10.1007/s00068-023-02402-w

**Published:** 2023-12-06

**Authors:** Thomas Mittlmeier

**Affiliations:** Department of Trauma, Hand and Reconstructive Surgery, Rostock University Medical Center, Rostock, Germany

Fractures of the tibia shaft are frequent with an incidence between 17 and 23 per 100,000 person-years [1]. Thus, they rank among the bread and butter business in trauma centers of any level. Correspondingly, all of us have developed our daily standards in managing these frequent fracture entities and our daily routine might make us doubt if there were any unmet needs or white spots in such a common fracture location. Maybe that the frequent handling of these fractures suggests that we have already achieved a level of optimized fracture care with highest degree of evidence which is hard to be improved. However, to anticipate this, there certainly are issues which need to be further addressed.

The present focus on tibia edition is not encyclopedical and refers to some selected topics and solutions which deserve closer attention and eventual implementation in our routine covering the field from preoperative diagnostics to peri- and intraoperative management and postoperative evaluation. Some of them as the relevance and assessment of tibial maltorsion, e.g., following closed intramedullary interlocking nailing after tibial fracture, had been studied decades ago [2, 3]. Furthermore, there had been an early proposal how to assess tibial torsion with high accuracy after closed intramedullary nailing of the tibia employing a 3-D ultrasound technique which did not gain much popularity, until now [4].

Now, an intraoperative standardized protocol based on a 2-D C-arm analysis of the a.p. projections of the knee and ankle of both legs pre- and intraoperatively offers the chance to relevantly reduce the risk for torsional malalignment in closed nailing of tibial shaft fractures. The method had been proven to offer a high degree of accuracy and reliability independent from the clinical experience of the surgeon [5].

Imaging is of paramount importance during analyzing and planning surgery for tibial shaft spiral fractures representing a low energy trauma in most cases. Plain X-rays allow for detection of 17% of the cases with fracture line extension into the ankle joint, only [6]. Even with awareness of this factor little more than 50% of the cases with concomitant fracture of the posterior malleolus are diagnosed relying on standard X-rays, Therefore, a CT scan should be performed preoperatively as a routine to avoid intraoperative complications as secondary fragment displacement [6].

Nowadays, we do rely on data from comparative studies preferably acquired in randomized controlled trials. Recently, it had been shown that the robustness of such trials in orthopaedic trauma is not that stable as we thought before where a limited number of event reversals might alter the trial’s significance [7]. Now, Minhas and coworkers [8] have analyzed in a systematic review that with respect to the non-union rate with and without reaming of the intramedullary canal in tibial nail fixation 87.5% of the studies did not find significant differences which is line with concurrent publications [9], but more than 78% of the findings were statistically fragile. This means that on average 2 event reversals for statistical findings and 4 event reversals for insignificant findings will suffice to alter statistical significance. Therefore, additional statistical metrics beyond the stereotypic *p* values should be introduced as the fragility index and quotient and the corresponding reverse fragility index and quotient to adequately describing this situation.

With regard to complex trauma more than every forth of tibial or lower leg fractures come along with a relevant soft tissue injury [1]. While it is widely accepted that a two-staged management with primary external fixation and early conversion to internal fixation within 14 days and final soft tissue or flap coverage within 7 days in type III A or IIIB open tibial fractures without increasing risk for complications Ma and coworkers have challenged this concept [10]. They had performed in a limited series of eight patients with III A and 7 III B open tibial fractures immediate one-stage debridement, reduction and internal fixation including flap coverage referring to the principles of enhanced recovery after surgery (ERAS) with favourable outcome. While there remain doubts concerning the correct primary assessment of the dynamics of severe soft tissue trauma and potential subsequent muscular necrotization their concept is near to the results and recommendations from Godina given in the mid-eighties [11] and needs further observation.

More than three decades ago there had been the assumption that scoring systems like the Mangled Extremity Severity Score (MESS) might aid the surgeon for decision-making in favor or against primary amputation. Unfortunately, the value of the MESS and similar scores did not prove true in many consecutive studies in civilian trauma. Gratl and coworkers focused their study on a large series of 50 patients with popliteal artery injury. MESS did not predict delayed amputation [12]. Hence, facing a limb survival rate of 88% they recommended that revascularization should always be attempted independent from any score.

Finally, after a mid-term follow-up following fracture of the tibial head outcome was not related to fracture type, injury severity or time from injury [13]. The most relevant functional limitations were found for sports and quality of life. Imbalances of gait were more evident during walking on stairs and outdoor walking on uneven ground than during ground level walking which could be objectivated via insoles. This underscores the role of wearables which are capable of monitoring gait function and the sequence of bony healing [14].

As such, our focus collection might entail a few steps forward towards clinical evidence in such a blockbuster topic as tibial fractures.

## References

[1] Mundi R, Chaudhry H, Niroopan G, Petrisor B, Bhandari M (2015). Open tibial fractures: updated guidelines for management. JBJS Rev.

[2] Strecker W, Keppler P, Gebhard F, Kinzl L (1997). Length and torsion of the lower limb. J Bone Joint Surg Br.

[3] Strecker W, Keppler P, Kinzl L (1998). Analysis of leg geometry. Langenbecks Arch Chir Suppl Kongressbd.

[4] Keppler P, Strecker W, Kinzl L, Simmnacher M, Claes L (1999). Sonographic imaging of leg geometry. Orthopäde.

[5] Bleeker NJ, Doornberg JN, ten Duis K, El Moumni M, Reininga IHF, Jaarsma RL (2023). Upma FFA on behalf of the traumaplatform 3D Consortium. Intraoperative fluoroscopic protocol to avoid rotational malalignment after nailing of tibia shaft fractures: introduction of the ‘C-arm rotational view (CARV)’. Eur J Trauma Emerg Surg..

[6] Lisitano L, Röttinger T, Wiedl A, Rau K, Helling S, Cifuentes J, Jehs B, Härting M, Feitelson L-M, Gleich J, Kiesl S, Pfeufer D, Neuerburg C, Mayr E, Förch S (2023). Plain x-ray is insufficient for correct diagnosis of tibial shaft spiral fractures: a prospective trial. Eur J Trauma Emerg Surg.

[7] Parisien RL, Dashe J, Cronin PK, Bhandari M, Tornetta P (2019). Statistical significance in trauma research: too unstable to trust?. J Orthop Trauma.

[8] Minhas A, Berkay F, Ehlers CB, Froehle AW, Krishnamurthy AB (2023). The statistical fragility of intramedullary reaming in tibial nail fixation: a systematic review. Eur J Trauma Emerg Surg.

[9] Flanagan CD, Vallier HA (2023). What’s new in orthopaedic trauma?. J Bone Joint Surg Am.

[10] Ma X, Wang Z, Wang J (2023). Clinical analysis of accelerated rehabilitation surgery for Gustilo type IIIA/B open tibio-fibular fracture. Eur J Trauma Emerg Surg.

[11] Godina M (1986). Early microsurgical reconstruction of complex trauma of the extremities. Plast Reconstr Surg.

[12] Gratl A, Kluckner M, Gruber L, Klocker J, Wipper S, Enzmann FK (2023). The mangled extremity severity score (MESS) does not predict amputation in popliteal artery injury. Eur J Trauma Emerg Surg.

[13] Gahr P, Mittlmeier T, Grau A, Herlyn PKE, Rahn A, Fischer D-C (2023). Functional assessment and outcome following surgical treatment of displaced tibial plateau fractures: a retrospective analysis. Eur J Trauma Emerg Surg..

[14] Warmerdam E, Orth M, Pohlemann T, Ganse B (2023). Gait analysis to monitor fracture healing of the lower leg. Bioengineering.

